# Effect of Final Thermomechanical Treatment on the Mechanical Properties and Microstructure of T Phase Hardened Al-5.8Mg-4.5Zn-0.5Cu Alloy

**DOI:** 10.3390/ma16083062

**Published:** 2023-04-13

**Authors:** Kai Tao, Jingbo Xu, Di Zhang, Aimin Zhang, Guang Su, Jishan Zhang

**Affiliations:** 1 School of Materials Science and Engineering, Henan Institute of Technology, Xinxiang 453003, China; 2 Engineering Research Center for Metallic Materials Modification Technology of Henan Province, Henan Institute of Technology, Xinxiang 453003, China; 3 State Key Laboratory for Advanced Metals and Materials, University of Science and Technology Beijing, Beijing 100083, China

**Keywords:** Al-Mg-Zn-Cu alloy, thermomechanical treatment, aging, hardness, tensile property, T phase

## Abstract

The effect of final thermomechanical treatment (FTMT) on the mechanical properties and microstructure of a T-Mg_32_(Al Zn)_49_ phase precipitation hardened Al-5.8Mg-4.5Zn-0.5Cu alloy was studied. The as-cold rolled aluminum alloy samples were subjected sequentially to solid solution treatment, pre-deformation, and two-stage aging treatment. Vickers hardness was measured during the aging process under various parameters. Tensile tests were conducted on the representative samples based on the hardness results. Microstructural characteristics were analyzed via transmission electron microscopy and high-resolution transmission electron microscopy. The conventional T6 process was also carried out for comparison. The hardness and tensile strength are increased evidently by the FTMT process for the Al-Mg-Zn-Cu alloy, while the ductility is adversely affected to a small extent. The precipitation at the T6 state consists of a coherent Guinier–Preston zone and T″ phase in the form of intragranular, fine, and spherical particles, while a semi-coherent T′ phase appears after the FTMT process as a new constituent. The distribution of dislocation tangles and isolated dislocations is another feature of FTMT samples. Enhanced precipitation hardening and dislocation strengthening account for the improved mechanical performance of FTMT samples.

## 1. Introduction

Aluminum alloys have been utilized increasingly in different transport sectors such as automobiles, airplanes, ships, and railway vehicles on account of their superior strength-to-weight ratio, formability, and corrosion resistance [1,2]. The well-received aluminum alloys so far consist of categories such as Al-Cu, Al-Mg, Al-Mg-Si [3,4], Al-Zn-Mg-Cu [5], etc. Deformation strengthening and precipitation hardening are the two significant strengthening mechanisms for aluminum alloys [6,7,8,9]. With respect to deformation strengthening, the effects of rolling and equal channel angular pressing have been widely studied. Precipitation hardening wise, composition modification together with a great diversity of heat treatment processes are investigated. In the meantime, novel types of aluminum alloy compositions are being designed and properties are being improved due to the sustainable effort from researchers worldwide.

Al-Mg alloys possess excellent formability, weldability, and corrosion resistance [10,11], and are extensively adopted in the automobile and marine industries [12,13,14,15]. However, the engineering performance still needs enhancement. According to recent reports [13,16,17,18,19,20], Sc, Zr, and Ag as the microalloying elements have demonstrated prominent improvement in Al-Mg alloy properties. However, there are probable obstacles in engineering applications considering the economic cost. Zn as an economical metal normally works as the primary strengthening element in 7xxx Al alloys, possessing the ability to form η phase. It has been found that the Al-Mg alloy with the addition of Zn as one of the alloying elements possesses higher strength and better intergranular corrosion resistance compared with traditional Zn-free alloy [12,21,22,23,24,25]. Differing from the phenomenon of Zn precipitating as η phase [26], all the precipitates are formed as T-Mg_32_(Al Zn)_49_ phase when the Zn/Mg ratio (wt.%) is within the range of ≤1.0 [8,10,24,27]. With the research progress in this direction, a novel group of precipitation-hardened aluminum alloys was designed and studied with Mg, Zn, with or without Cu as the primary alloy elements, referred to as the AlMg/AlZnMg(Cu) crossover alloys [28], featured by the composition ratio (wt.%) of Zn/Mg ≤ 1.0 and Cu/Mg ≤ 0.5 [8,27,29]. It was found that mechanical properties and intergranular corrosion resistance rise with the increase in the (Zn + Cu)/Mg ratio [27]. In a recent study, it was found that the strengthening effect of T phase precipitation was not well achieved under the T6 process for an Al-Mg-Zn-Cu alloy with a Zn/Mg ratio less than 0.8 [27].

With the purpose of further improving the performance of Al-Mg-Zn-Cu alloy, thermomechanical treatment was considered, which is well-received in the heat treatment of aluminum alloys [18,30,31]. Based on the mechanisms of precipitation promotion, it can be further classified into intermediate thermomechanical treatment (ITMT) and final thermomechanical treatment (FTMT) [32], and the latter is the case in this paper. The FTMT process was studied on the Al-5.8Mg-4.5Zn-0.5Cu alloy, with Zn/Mg ≈ 0.78 and Cu/Mg ≈ 0.086; meanwhile, a two-stage aging process was also carried out as a counterpart, trying to find out the effect of FTMT process on the mechanical properties and microstructure of Al-Mg-Zn-Cu alloy.

## 2. Materials and Methods

The nominal chemical composition of the experimental Al-Mg-Zn-Cu alloy is listed in Table 1. The samples were prepared by induction furnace melting and then chill casting in a steel mold with circulating water. The ingot was sequentially subjected to homogenization treatment, milling, hot rolling, recrystallization treatment, and cold rolling, with a final thickness of 2.5 mm.

**Figure 1:**
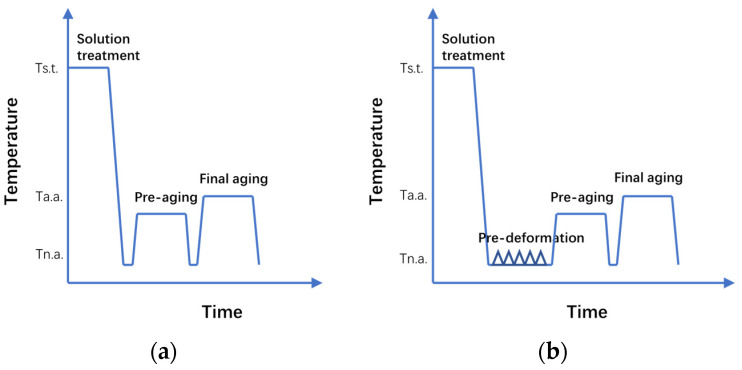
The schematic illustrations of processing routes for the Al-Mg-Zn-Cu alloy after cold rolling: (**a**) T6 route; (**b**) FTMT route.

The as-cold rolled samples were solid solution treated at 480 °C for 30 min in an air circulation furnace followed by immediate water quenching. Then the samples were separated into two groups. One group was directly subjected to the peak aging (T6) process; the other went through the FTWT process with a pre-deformation (cold rolling) extent of 5–10%. The two-stage aging process has demonstrated superior mechanical properties to a single aging process for this series of alloys [33,34] and was adopted in this study to achieve a good peak aging effect for both groups of samples. The schematic illustrations of the two processing routes for Al-Mg-Zn-Cu alloy after cold rolling are shown in Figure 1.

The hardness measurements were conducted via an HVST-1000Z micro-Vickers hardness tester (Shanghai Ju Hui Instrument Manufacturing Co., Ltd., Shanghai, China) on the transverse section parallel to the longitudinal direction, with a load of 200 g and dwelling time of 15 s. Each presented hardness value is the average of 7 individual measurements. The tensile specimens were taken in the rolling direction with a gauge length of 25 mm and a width of 6 mm as per the ASTM E8/E8M-11 standard [35]. The tensile tests were performed on a CMT4105 universal testing machine at room temperature, and each reported tensile value is the average of 3 individual tests.

The foil samples for transmission electron microscopy (TEM) observation were thinned by twin jet electro-polishing on the studied alloy disks with a diameter of 3 mm, at temperatures between −30 °C and −35 °C. The jet solution consisted of 25 vol% nitric acid and 75 vol% methanol. TEM and high-resolution transmission electron microscopy (HRTEM) analyses were carried out with the JEOL-2010 and FEI Tecnai-F20 instruments. Image-Pro Plus analysis software (Version 6.0) was used to facilitate the calculation of the mean diameter and volume fraction of precipitates after different treatments.

## 3. Results

### 3.1. Hardness and Tensile Properties under the T6 Treatment

Based on the previous experiment results [21,33] and the DSC analysis, 480 °C was selected as the solution treatment temperature with a holding time of 30min. The parameters for the pre-aging and final aging stages were set as 90 °C/24 h + 140 °C/25 h and 90 °C/48 h + 140 °C/13 h, respectively, with reference to optimized conditions in early studies [21,36], denote as T6-a and T6-b. as listed in Table 2 with the detailed process information. The hardness values were measured to be 173.54 and 175.76 HV0.2 correspondingly.

**Figure 2:**
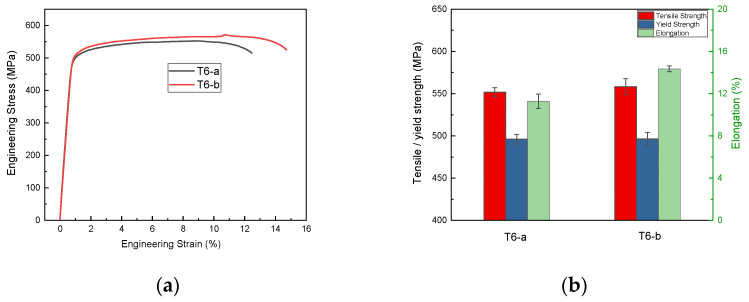
Stress–strain curves and tensile properties of Al-Mg-Zn-Cu alloy after T6 process with different parameters: (**a**) stress–strain curves; (**b**) tensile properties.

Tensile tests were carried out on the T6-a and T6-b samples, and the stress–strain curves are shown in Figure 2a. The tensile results are consistent with the hardness test, where the two samples possess similar mechanical properties. The T6-b sample exhibits better tensile properties, with a tensile strength of 558.3 MPa, yield strength of 496.4 MPa, and elongation of 14.35%, as seen in Figure 2b.

### 3.2. Hardness and Tensile Properties under the FTMT Process

**Figure 3:**
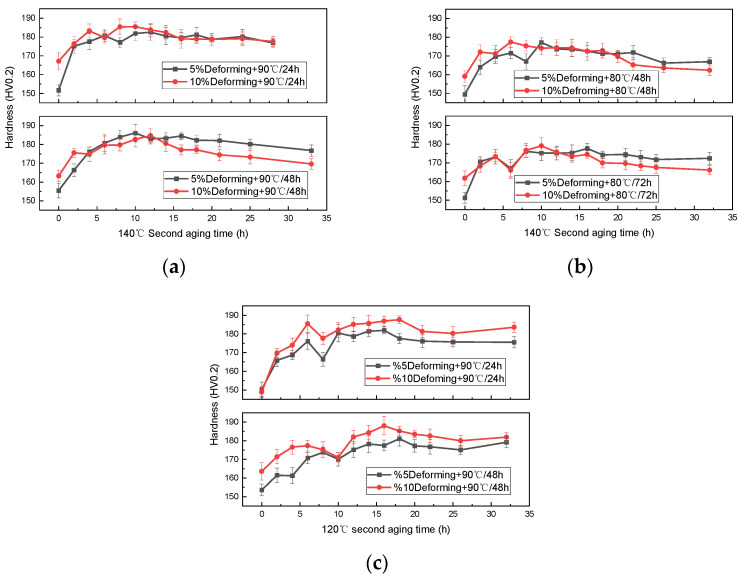
Hardness variation of the FTMT samples with different final aging times: (**a**) pre-deformation + pre-aging at 90 °C + final aging at 140 °C; (**b**) pre-deformation + 80 °C + 140 °C; (**c**) pre-deformation + 90 °C + 120 °C.

The pre-deformation extent adopted for the FTMT process in this study varied between 5–10%. Based on the previous results [30,32], the peak aging process parameters for FTMT are close to the T6 treatment, thereby some parameters from T6-a and T6-b processes were selected as the starting configuration. The hardness variation curves with final aging time for the FTMT samples pre-aged at 90 °C and final aged at 140 °C are presented in Figure 3a. For comparison convenience, all the scale ranges of the vertical axis in the graphs related to the hardness and tensile values hereafter are purposely set unified. The hardness development of the four samples demonstrates a similar trend to some degree, which can be classified into 4 stages: (1) the hardness rises quickly during the initial several hours, due to the forming and growth of the precipitated phase; (2) then the value drops within a small extent, mainly caused by the reduction of dislocation density in the sample; (3) after that there is an obvious ascending to the maximum, sometimes with little fluctuations, indicating that the matching between recovery and precipitation strengthening reaches an optimal state at this stage; and (4) finally, the hardness decreases slightly and develops into a relatively stable state, which means the change of dislocation density also reaches a relatively steady state in the over-aged alloy matrix. Neither the variation in deformation extent nor the pre-aging time shows an obviously dominant influence on the hardness evolution. The hardness variation depends mainly on the mutual influence and interaction of precipitation strengthening and dislocation strengthening effects in the final aging process. The 4 groups of processes yield close hardness values, with a maximum of around 186 HV0.2 (185.5 and 186.1 HV0.2 for the hardness lines marked in black and red, respectively) after final aging for 10 h, which demonstrate hardness improvement compared with the T6 process.

In consideration of the extra internal energy generated during the pre-deformation process, lower aging temperature could be more suitable for the FTMT samples, therefore the temperatures of the two-stage aging process were optimized towards the lower side. Figure 3b shows the hardness development with final aging time at 140 °C after pre-aging at 80 °C for 48 h or 72 h for the FTMT samples. The two sample groups with 10% pre-deformation both exhibit higher initial hardness due to the deformation strengthening effect. The variation trends during the final aging period generally share the four-stage characteristics, similar to the samples shown in Figure 3a. Under the condition of pre-aging at 80 °C and final aging at 140 °C, the maximum hardness value is about 180 HV0.2 (179.06 and 180.72 HV0.2 for the hardness lines marked in black and red, respectively). The general hardness level is decreased compared with the pre-aging temperature of 90 °C. The reason is probably that insufficient fine precipitation phases (GP zones) formed during the pre-aging stage [33,37] under the lower temperature of 80 °C, which results in weaker precipitation hardening effects in the final aging period.

Following the idea of lowering the aging temperature, another four groups of Al-Mg-Zn-Cu samples were FTMT treated with a pre-deformation extent of 5% or 10%, then pre-aging at 90 °C for 24 h or 48 h, and final aging at 120 °C. The corresponding hardness variation with final aging time is exhibited in Figure 3c. The above-mentioned four-stage hardness evolution features with final aging time were clearly presented in this scenario, however, with longer periods to reach the maximum value. Moreover, the effect of a larger pre-deformation extent on the hardness improvement was distinctly demonstrated during the entire final aging period. The maximum hardness achieved under this condition is around 188 HV0.2 (187.64 and 188.02 HV0.2 for the hardness lines marked in black and red, respectively), with an 8% increase compared with the best result of the T6 process.

To further verify the optimized FTMT process parameters for the Al-Mg-Zn-Cu alloy, representative samples from each set of process groups, corresponding to the highest two hardness points from Figure 3a–c, respectively, were carried out by tensile test following the AETM E8/E8M-11 standard. The samples tested were denoted, as listed in Table 3 with their primary process parameters.

**Figure 4:**
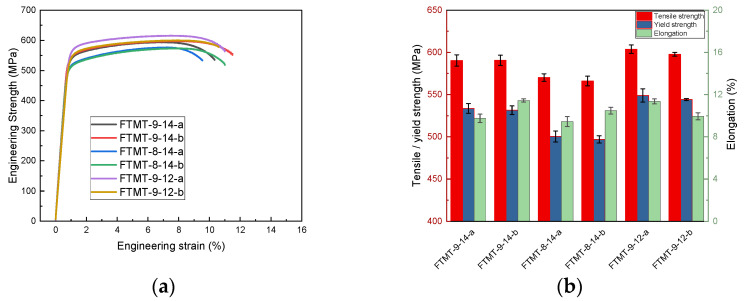
Stress–strain curves and tensile properties of Al-Mg-Zn-Cu alloy after FTMT process with different parameters: (**a**) stress–strain curves; (**b**) tensile properties.

The stress–strain curves and tensile properties of the FTMT samples are shown in Figure 4. The tensile strength results are generally consistent with the hardness measurement, among which FTMT-9-12-a with solution treatment at 480 °C for 30 min, pre-deformation of 10% extent, pre-aging at 90 °C for 24 h, and final aging at 120 °C for 18 h possesses the best mechanical property combination, with a tensile strength of 603.7 MPa, yield strength of 549.0 MPa and elongation of 11.36%, as displayed in Figure 4b. Under the optimized FTMT process, tensile strength was increased by 45.4 MPa and yield strength increased by 52.6 MPa, while the elongation rate was slightly decreased by 2.99% compared with the T6 process. The 6 samples of the FTMT processes exhibit a narrow mechanical property distribution, which means the studied Al-5.8Mg-4.5Zn-0.5Cu alloy has good processing properties, and will facilitate the potential industrial applications.

### 3.3. Microstructural Characteristics

**Figure 5:**
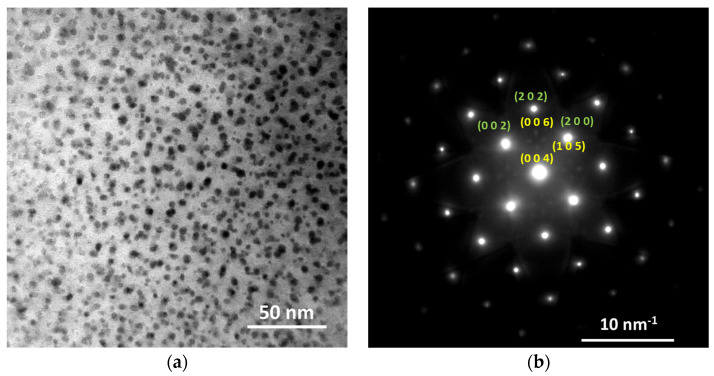
TEM images showing the typical microstructure of T6–b sample: (**a**) BF image; (**b**) SAED pattern.

The microstructure of representative samples under different processes was analyzed via TEM and HRTEM. The bright field (BF) TEM image of the T6-b sample is displayed in Figure 5a, where fine and globular precipitates with an average diameter of several nanometers are dispersed uniformly in the aluminum alloy matrix. The corresponding selected area electron diffraction (SAED) pattern is shown in Figure 5b. The characteristic dot pattern of the aluminum alloy matrix is identified and marked with green notes. While the relatively weak dot pattern, two dots of which are located at the positions of 2/5 and 3/5 length of (202) Al, is identified as the T phase marked with notes in yellow.

**Figure 6:**
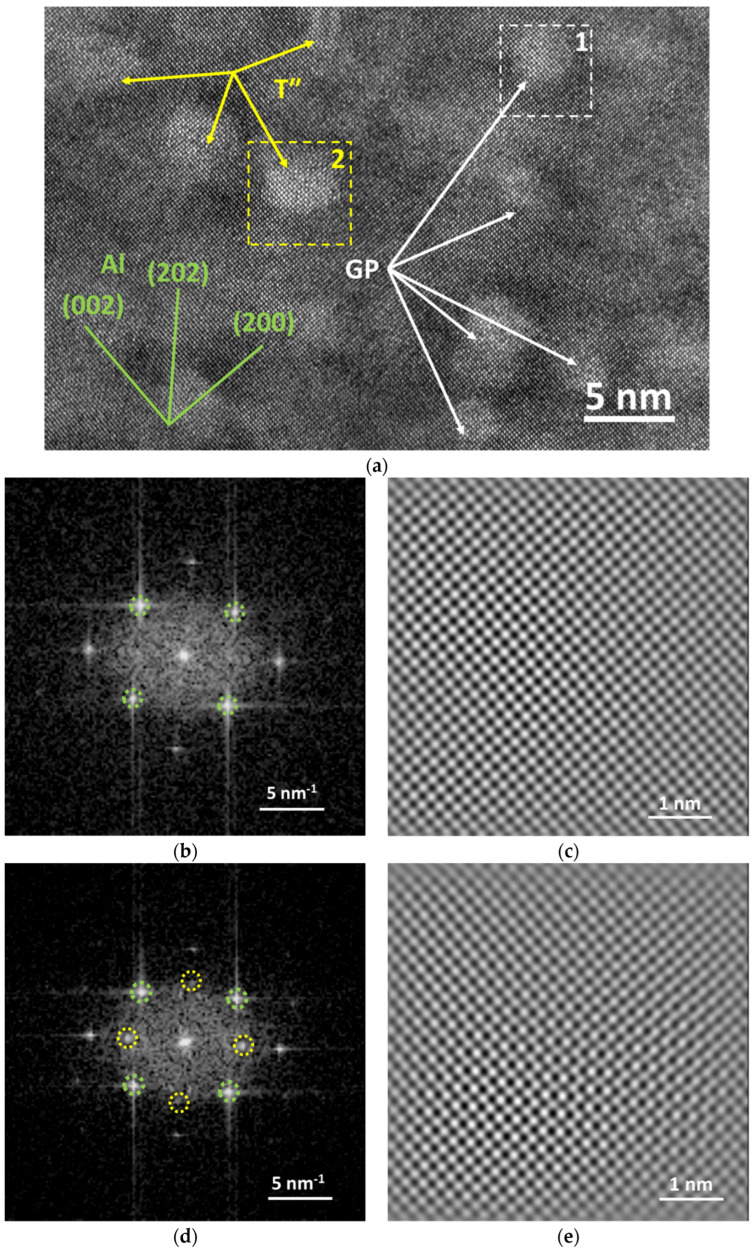
HRTEM images of T6–b sample: (**a**) typical HRTEM image; (**b**) FFT pattern of square zone 1; (**c**) IFFT image generated from the pattern in (**b**); (**d**) FFT pattern of square zone 2; and (**e**) IFFT image generated from the pattern in (**d**).

The typical HRTEM image of the T6 sample is displayed in Figure 6a. Some roughly spherical precipitates are dispersed in the aluminum matrix, as indicated by arrows. The two in the positions of colored squares with numbers are Fourier transformed, as shown in Figure 6b,d. Two types of fast Fourier transformation (FFT) patterns are found. One is identified as only the aluminum alloy phase, marked with green circles; while the other is identified as the aluminum alloy phase and Mg_32_(Al Zn)_49_ T phase, marked with green and yellow circles, respectively, consistent with the SAED result from TEM analysis. The inverse fast Fourier transform (IFFT) images are generated from the dots marked in the FFT pattern, as shown in Figure 6c,e. Although lattice differences can be found in the areas of two precipitates, the coherency relations both show a full-coherence feature. Based on the TEM-SAED pattern and HRTEM-FFT results, it can be concluded that the microstructure of T6 sample comprises 3 phases: aluminum alloy matrix, Guinier–Preston (GP) zone, and T″ phase [8]. The two types of precipitates are marked with white and yellow arrows, respectively, in Figure 6a, while the lattice planes of the aluminum alloy matrix are marked with green bars and indices.

**Figure 7:**
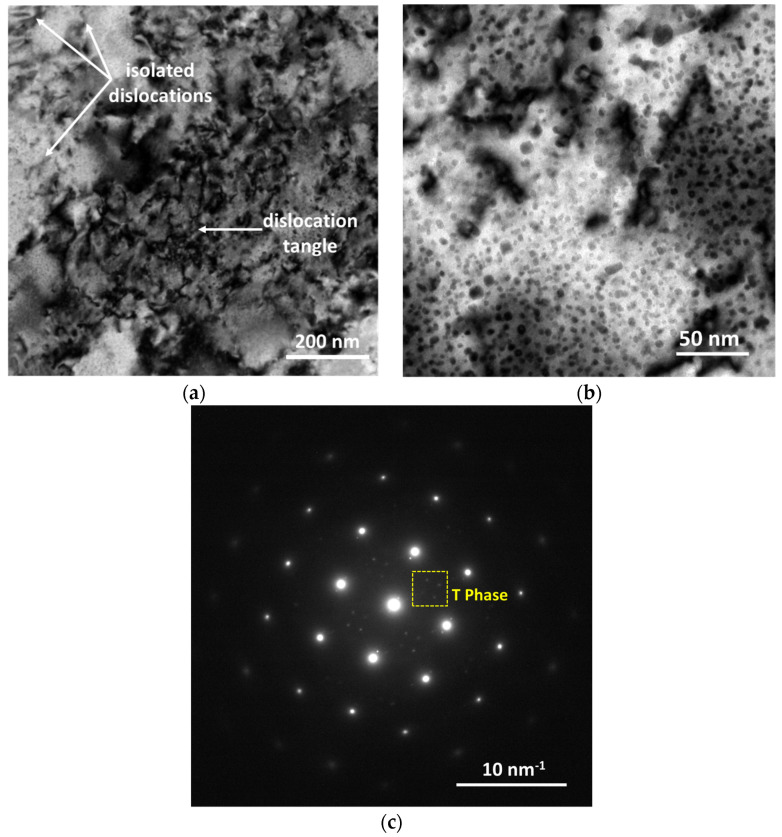
TEM images showing the typical microstructure of FTMT–9–12–a sample: (**a**) BF image showing the dislocation distribution; (**b**) BF image showing the precipitates; and (**c**) SAED pattern corresponding to (**b**).

The TEM images of the FTMT–9–12–a sample are displayed in Figure 7. The BF image in Figure 7a shows the dislocation distribution, where some dislocation tangles and isolated dislocations can be identified, marked by arrows. The BF image of higher magnification in Figure 7b shows the precipitate morphology under the FTMT process, where fine and roughly globular precipitates with relatively uniform size are dispersed in the aluminum alloy matrix. The SAED pattern corresponding to Figure 7b is shown in Figure 7c. Similar to the situation in Figure 5b, the characteristic dot pattern of the T phase is identified beside the aluminum matrix phase, which is marked with a yellow square in the image.

**Figure 8:**
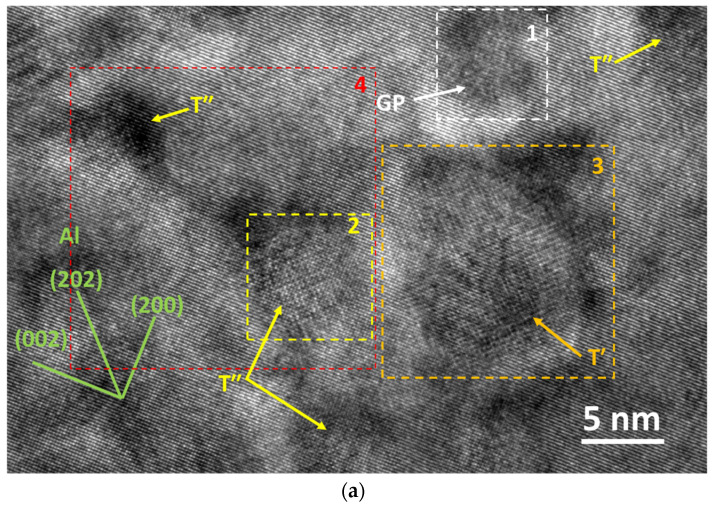

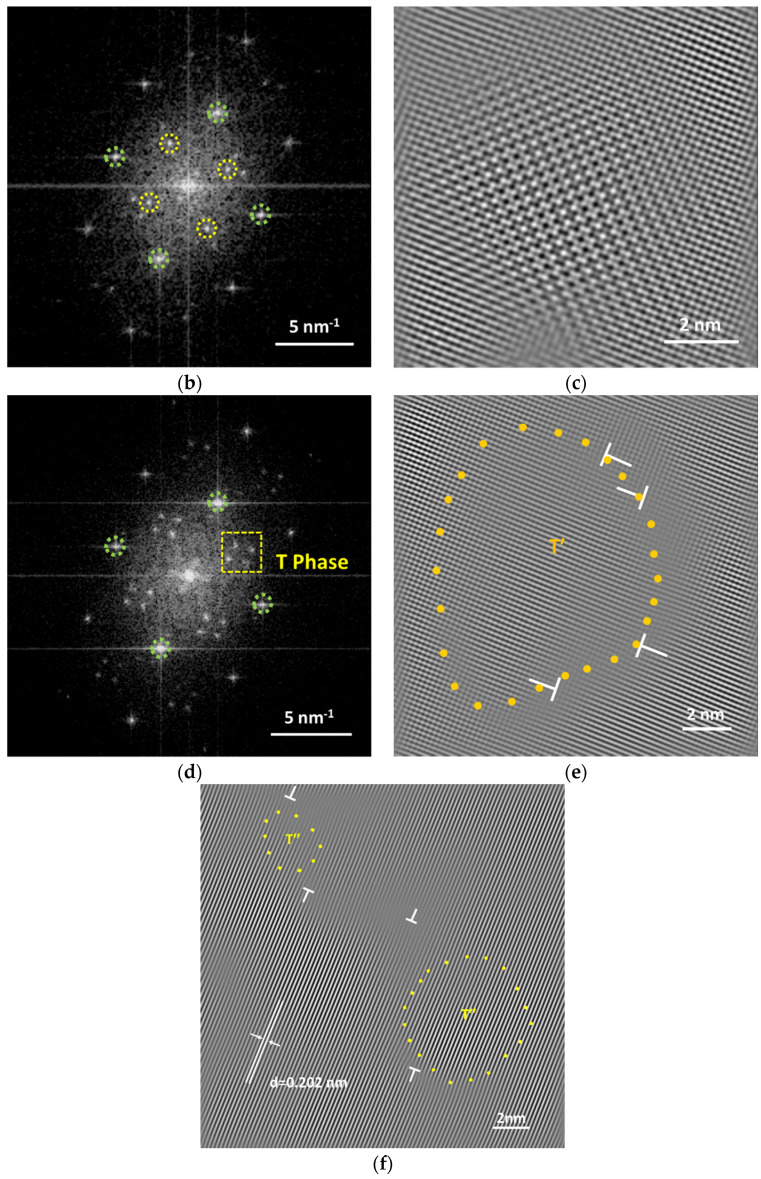
HRTEM images of FTMT–9–12–a sample: (**a**) typical HRTEM image; (**b**) FFT pattern of square zone 2; (**c**) IFFT image generated from the pattern in (**b**); (**d**) FFT pattern of square zone 3; (**e**) IFFT image generated from the pattern in (**d**); and (**f**) IFFT image corresponding to square zone 4.

The typical HRTEM image of the FTMT sample is shown in Figure 8a. Roughly globular precipitates can be found in the metallic matrix, and FFT operations were conducted on these precipitation regions. The characteristic FFT patterns of GP zones and T-series phases are identified. The result is similar to the scenario for the T6 sample, except that the GP zone seems less in proportion. When IFFT was conducted on these areas, however, some results show distinctive features compared with the T6 counterpart. Figure 8b displays the FFT pattern of square zone 2, while Figure 8c shows the IFFT image generated from the pattern dots in Figure 8b, which were marked with green and yellow circles, corresponding to the aluminum alloy phase and T-series phase, respectively. The phase in zone 2, with a diameter of around 5 nm, was identified as T″ phase for its full-coherent relation with the matrix, but high lattice strain is clearly presented in the IFFT image. The precipitate in square zone 3 possesses a relatively large size of around 9 nm, and its FFT pattern is displayed in Figure 8d, which consists of characteristic dots corresponding to the aluminum alloy phase (marked with green circles) and the T-series phase (marked with a yellow square). The IFFT image generated from the dots marked by green circles in Figure 8d is shown in Figure 8e, where several edge dislocations can be identified, marked with the symbol of white cross bars. Thus, this precipitate with a larger size is identified as T′ phase [8,24] for its semi-coherent lattice relation with the metallic matrix [21].

Figure 8f displays the IFFT image corresponding to zone 4, marked with a red square. The crystalline interplanar spacing is measured as 0.202 nm, corresponding to the aluminum alloy matrix. Several edge dislocations are distributed in the matrix, near which some fine and spherical precipitates are formed. This can be explained as the extra energy induced by the dislocations facilitating the formation of the T-series phase precipitates. The microstructural feature here is well consistent with the TEM images. Based on the TEM and HRTEM results, it can be concluded that the phase constituent of the FTMT sample consists of 4 phases: aluminum alloy matrix, GP zone, T″ phase, and T′ phase. The precipitates are marked with arrows of different colors, while the lattice planes of the aluminum alloy matrix are marked with bars and indices in green, as seen in Figure 8a.

## 4. Discussion

### 4.1. The Microstructure and Mechanical Properties of Al-Mg-Zn-Cu Alloy under FTMT Process

The mechanical properties of the Al-Mg-Zn-Cu alloy sample are effectively improved by the FTMT process compared with the T6 process. The main reason could be attributed to the effects of dislocation strengthening and enhanced precipitation hardening. The pre-deformation in the FTMT process introduces a number of dislocations in the metallic matrix. The typical morphology of dislocations comprised mainly dislocation tangles and isolated dislocations. No obvious dislocation cell or sub-grain structure is found under the TEM analysis, which is different from other aluminum alloys under the TMT process [7,18,30], probably due to the relatively small deformation extent during the rolling process before the two-stage aging.

**Figure 9:**
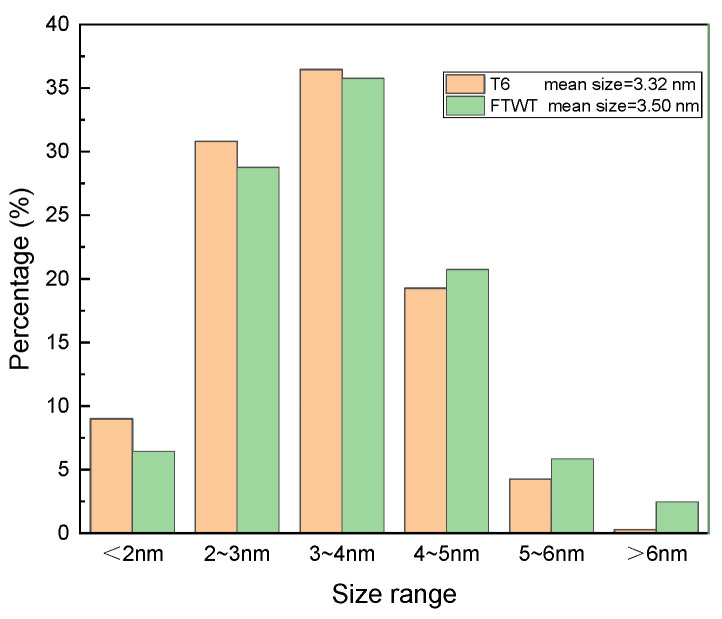
The distribution for the sizes of precipitated phases under different processes.

To evaluate the contribution of precipitation hardening, the size distribution of the precipitation phases was measured from the BF-TEM images by means of the software. The thickness of the statistical region was set as 70 nm [21], then the volume fraction could be obtained by the total volume of precipitates dividing the volume of the statistical region, with the simplified assumption that the shape of phases is sphere-like. The results indicate that the mean diameter of the precipitation phase is 3.32 nm and the volume fraction of the precipitations is 0.76% for the T6-b samples; while the mean diameter is 3.50 nm and the volume fraction of the precipitations is 0.82% for FTMT samples. All the above data are the statistical results from measurements of more than 700 precipitates and the corresponding areas. The distribution for the diameters of precipitated phases under different processes is exhibited in Figure 9, where the tendency towards the larger size region is clearly demonstrated for the FTMT sample compared with its counterpart under the T6 process. The extra energy which was introduced mainly by dislocations generated during the pre-deformation process should account for the larger mean size and volume fraction of precipitated phases under the FTMT process.

Based on the dispersion strengthening mechanism, the influence of precipitation characteristics on the tensile strength of aluminum alloy can be described as Function (1) [20,22]:

(1) $$\Delta \sigma_{p}=M\chi {\left(\varepsilon G\right)}^{3/2}{\left(\frac{rf}{0.5Gb}\right)}^{1/2}$$

where $\Delta \sigma_{p}$ is the precipitation strengthening induced increase in yield strength; M corresponds to the Taylor factor with the value of 2.6 based on the Hutchinson model [38]; the value of 2.0 for the constant coefficient χ is adopted here [22]; ε is the misfit parameter with the value of about 3.53 [22,24]; G is the shear modulus with the value of 26 GPa for Al alloy; r stands for the mean diameter of precipitated particles; f stands for the volume fraction of the T-series precipitates; and b is the Burgers vector adopted as 2.84 Å for Al alloy. Based on the above-mentioned numbers, $\Delta \sigma_{p}$ are calculated according to Function (1), as listed in Table 4.

The contribution percentages of precipitation hardening to the yield strength are calculated to be 76.4% and 73.6% for the T6 and FTMT samples, respectively. The result is very comparable to that from the previous report of Al-5.6Mg-3.1Zn-0.15Cu alloy [22] after peak aging treatment, where the $\Delta \sigma_{p}$ contribution is around 80.3% for the yield strength. Considering the extra deforming strengthening for the FTMT sample, the lower contribution percentage is reasonable. Among the strengthening factors of solid solution, grain boundary, precipitation, and dislocation, precipitation strengthening plays the dominant role for the FTMT samples of Al-Mg-Zn-Cu alloy. According to the calculated result, the larger value of r and f for the FTMT sample do contribute to the strength increase.

**Figure 10:**
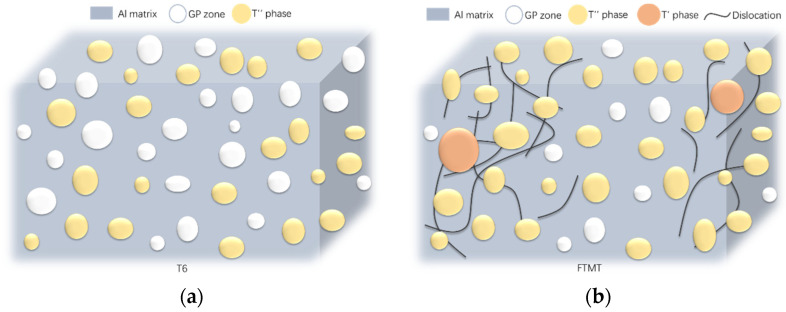
Illustration of intragranular microstructure for Al-Mg-Zn-Cu alloy under different processes: (**a**) T6 process; (**b**) FTMT process.

The sketch models of intragranular microstructure for Al-Mg-Zn-Cu alloy under T6 and FTMT processes are illustrated in Figure 10. Compared with the T6 sample, the microstructure of the FTMT sample is featured by the formation of the T′ phase and uneven distribution of isolated dislocations and dislocation tangles, besides the common feature of GP zone, and T” phase. Moreover, the pre-deformation promotes not only the new phase formation but also the growth of precipitate size. The conjunction effect of the aforementioned reasons results in the better mechanical performance of the FTMT samples.

### 4.2. The Practical FTMT Process

There are generally two types of FTMT processes for the Al-Mg-Zn-Cu alloy. The main difference lies in the stage sequence of pre-deformation during the entire process. For the first type, the pre-deformation is performed after the pre-aging stage [18,39], which was adopted in the earlier studies of our group [10,22,36]. The advantage of this arrangement is that artificial aging can be initiated quickly after the solid solution treatment, thus the potential negative effect of natural aging [21] during the stage intervals of the process can be mitigated. However, we were recently informed by our cooperative partner from the plant that the pre-deformation could be difficult to carry out due to the hardness rising after the pre-aging process. Therefore, the second type of FTMT process for Al-Mg-Zn-Cu alloy, featuring pre-deformation before the pre-aging, was experimented with in this study for better adaptation to the industrial application. The typical microstructural characteristics of the two FTMT processes are similar, and the mechanical properties are comparable [22,27], which means strong adaptability of the FTMT process for the Al-Mg-Zn-Cu alloy production. In addition, the relatively small variations of hardness and tensile properties between different process parameters demonstrate good processing properties of the Al-Mg-Zn-Cu alloy. These characteristics all facilitate the application of Al-Mg-Zn-Cu alloy in industrial production conditions.

## 5. Conclusions

The mechanical properties and microstructure of T phase precipitation hardened Al-5.8Mg-4.5Zn-0.5Cu alloy were studied under the FTMT process.

(1) The as-cold rolled aluminum alloy samples were subjected sequentially to solid solution treatment, pre-deformation, and two-stage aging treatment. Vickers hardness was measured during the final aging process under various parameters. The maximum hardness achieved is around 188 HV0.2, with an evident increase compared with the T6 process.
(2) Tensile test results are consistent with the hardness measurements. The ultimate and yield tensile strength are increased effectively by the optimized FTMT process, while the ductility is adversely affected to a small extent. The best properties achieved are 603.7 MPa, 549.0 MPa, and 11.36% for the tensile strength, yield strength, and elongation, respectively.
(3) The phase constituent of the FTMT sample comprises four phases: aluminum alloy matrix, GP zone, coherent T″ phase, and semi-coherent T′ phase, while the precipitated particles at the T6 state consist of only GP zone and T″ phase.
(4) The typical morphology of dislocations for the FTMT sample comprises mainly dislocation tangles and isolated dislocations. The mean size and volume fraction of precipitated phases is increased under the FTMT process compared with the T6 process. Dislocation strengthening together with enhanced precipitation strengthening account for the improved mechanical performance of FTMT samples.
(5) The process parameter variation has limited effects on the mechanical properties of the Al-Mg-Zn-Cu alloy, which facilitates the application in the industrial production condition.

## Figures and Tables

**Table 1:** The nominal chemical composition of Al-5.8Mg-4.5Zn-0.5Cu alloy (wt.%).

Mg	Zn	Cu	Mn	Cr	Ti	Zr	Al	Zn/Mg	(Zn + Cu)/Mg
5.8	4.5	0.50	0.10	0.04	0.06	0.12	Balance	0.78	0.86

**Table 2:** Process stages and parameters for Al-5.8Mg-4.5Zn-0.5Cu alloy samples under the T6 process.

Sample	Solid Solution Treatment	Pre-Aging	Final Aging
T6-a	480 °C/30 min	90 °C/24 h	140 °C/25 h
T6-b	480 °C/30 min	90 °C/48 h	140 °C/13 h

**Table 3:** Process stage and parameters for Al-5.8Mg-4.5Zn-0.5Cu alloy samples under different processes.

Sample	Pre-Deformation Extent	Pre-Aging	Final Aging
FTMT-9-14-a	10%	90 °C-24 h	140 °C-10 h
FTMT-9-14-b	5%	90 °C-48 h	140 °C-10 h
FTMT-8-14-a	10%	80 °C-72 h	140 °C-10 h
FTMT-8-14-b	5%	80 °C-72 h	140 °C-16 h
FTMT-9-12-a	10%	90 °C-24 h	120 °C-18 h
FTMT-9-12-b	10%	90 °C-48 h	120 °C-16 h

**Table 4:** The mean diameter and volume fraction of precipitates for Al-Mg-Zn-Cu alloy samples under different processes.

Sample	*r* (nm)	*f* (%)	Δ*σ_p_* (MPa)
T6	3.32	0.762453	379.1852
FTMT	3.50	0.822041	404.2561

## Data Availability

Not applicable.
